# Full-Field Mapping and Flow Quantification of Melt Pool Dynamics in Laser Powder Bed Fusion of SS316L

**DOI:** 10.3390/ma14216264

**Published:** 2021-10-21

**Authors:** Asif Ur Rehman, Fatih Pitir, Metin Uymaz Salamci

**Affiliations:** 1 ERMAKSAN, 16065 Bursa, Turkey; Fatih.Pitir@ermaksan.com.tr; 2 Department of Mechanical Engineering, Gazi University, 06570 Ankara, Turkey; msalamci@gazi.edu.tr; 3 Additive Manufacturing Technologies Application and Research Center-EKTAM, Gazi University, 06560 Ankara, Turkey; 4 Manufacturing Technologies Center of Excellence-URTEMM A.S., 06980 Ankara, Turkey

**Keywords:** multi-physics model, LPBF process, multiple reflection, selective laser melting, selective laser sintering, Marangoni flow, metal, SS316L

## Abstract

Laser powder bed fusion (LPBF) has a wide range of uses in high-tech industries, including the aerospace and biomedical fields. For LPBF, the flow of molten metal is crucial; until now, however, the flow in the melt pool has not been described thoroughly in 3D. Here, we provide full-field mapping and flow measurement of melt pool dynamics in laser powder bed fusion, through a high-fidelity numerical model using the finite volume method. The influence of Marangoni flow, evaporation, as well as recoil pressure have been included in the model. Single-track experiments were conducted for validation. The temperature profiles at different power and speed parameters were simulated, and results were compared with experimental temperature recordings. The flow dynamics in a single track were exposed. The numerical and experimental findings revealed that even in the same melting track, the melt pool’s height and width can vary due to the strong Marangoni force. The model showed that the variation in density and volume for the same melting track was one of the critical reasons for defects. The acquired findings shed important light on laser additive manufacturing processes and pave the way for the development of robust, computational models with a high degree of reliability.

## 1. Introduction

Additive manufacturing (AM) offers customizable design, decreased processing time, and the possibility of creating complex geometries. It has garnered a lot of attention from advanced technology applications [1], aerospace [2], biomedical [3,4], and construction [5,6]. Laser powder bed fusion (LPBF) is among the most prominent AM innovations with prevalent benefits, considerably reduced structural constraints, good repeatability, and timely delivery [7]. In the LPBF, the metallic powder is deposited layer after layer through the blade or roller, followed by the fusion of particles through the laser on specific areas to create the required slices, driven by CAD data [8]. Several defects, such as balling, cracks, pores, or low-layer uniformity, are counterproductive to efficiency and part quality [9,10,11]. Consequently, the deformations and the effect of input variables on the melt pool need to be better understood [12,13].

It is noted that many variables affect the melt pool, and indirectly, the performance of the components manufactured [14], including scanning velocity, laser power, particle size distribution (PSD), and layer thickness [15]. Concerning the effecting process variables, systematic efforts were made to explain the complicated melt pool dynamics [12,16,17,18,19], process parameters, and frequent defects. The impact of laser power and scan velocity on the surface morphology of LPBF components was studied by Hodge et al. and Lin et al. [20,21]. Irregularities, deformations, cracks, and other defects on the surfaces are created at a faster scan rate, as per the analysis [20,21]. Another study concentrated on the development of the defect during the LPBF processes of the metallic powder particles [22]. The analysis showed that the energy density (ED) had quite a significant impact on the growth of defects. The explanation of physics behind the dynamic relationship between the process parameters is, nevertheless, insufficient. It is expensive and complicated to focus entirely on the Hit-and-Trial approach to retrieve the correct process variables throughout the LPBF studies. Moreover, the observational analysis [23,24] questions the various complex laws identified in the LPBF method. 

In the past, investigations concentrated mainly on dynamics and defect generation processes utilizing a powder-scale mesoscopic framework. Frazier et al. created a powder method that strongly maps the powers’ interface, including surface tension, Marangoni tension, and recoil [25], and the analysis indicated that the powder bed’s thickness could lead to voids. However, it must be mentioned that the closely packed powder bed is not the same as the actual power bed deposition and the distribution of particle sizes. Modelling the process of producing powder beds close to experiments is a precondition for a powder-scale LPBF simulation used in the Computational Fluid Dynamics (CFD) system, where the spreading method primarily includes deposition of the powder layer with the powder PSD of the given metallic alloys. To achieve that, the discrete element method (DEM) is often used to simulate the process of metallic powder particle deposition because of the associated physics. Atallah et al. utilized the DEM coupled with the finite volume method (FVM) [26] to examine the effects of the scanning velocity, power, and particle size on the melted region. Its analysis suggested that the balling defect arose from a higher scan rate and low beam power. To investigate the creation process of persistent irregularities during the LPBF process, Colosimoet al., Matthews et al., and Lee et al. [20,27,28] suggested a comparatively extensive model taking into account recoil pressure and the Marangoni effect. Their framework elucidated the scattering, pore defect-forming process. In addition, the analysis shows the critical influence of the recoil and the Marangoni force. Yan et al. and Khairallah et al. [29,30,31] have developed a multi-physics field framework to define single/multi-track defects’ creation for the Electron Beam Powder Bed Fusion (E-PBF) system. They observed how energy intake and the depth of the metallic powder particle greatly impacted the balling phenomenon. The powder selection and thickness of the metallic powder bed were two critical factors in determining the non-uniformity of its single track. Zeng et al., Everson et al., and Qian et al. explored the effects of process variables on the surface [32,33,34]. They noted that scanning velocity was a crucial parameter correlated strongly with the molten pool measurements and the surface morphology. Zhou et al., Everitt et al., and Tang et al. developed a physics-dependent CFD model [30,31,32], using the beam-tracing technique. The results reveal that the instability of the melt pool and keyhole triggered the generation of the voids. Most of the computational models listed above focus primarily on exploring the effect of different parameters [15,35,36,37,38,39]. In recent years, extensive studies have centered on the production of defects during the LPBF process [18,19,40]. However, there is still a lack of a thorough explanation of the melt pool mass flow migration and mass flow rate. The understanding of the root causes of these defects, including changes in phases, melt pool inconsistency, flow within the melt pool, and material density change (contributing to defect generation), needs to be explored in mesoscale so that possible inconsistencies between theory and experiment could be explained [38,41,42]. The LPBF process involves rapid melting and solidification, which is affecting all the thermo-physical properties involved [43]. A detailed material properties’ simulation is the foundation of the LPBF process simulation of the powder bed. Stainless-steel 316L (SS316L) has been employed in this investigation as the testing material. It has a medium chromium content as well as a high nickel concentration, which leads to good weldability and is indeed a significant advantage of SS316L in LPBF. This advantage is due to the low carbon and medium molybdenum concentration, which makes it resistant to hot and cold cracking [44,45,46,47]. 

In this work, we devised a method for tracking the melt flow behavior across the melt pool in LPBF. We determined the melt flow dynamics of the whole melt pool and studied the liquid flow and physical processes occurring inside it. We explain the inconsistent melt pool width of the track with the quantification of the mass flow rate. Moreover, the corresponding flow driven by the Marangoni effect is explained through the mass flow rate. In this study, the mass flowing forward and then being pulled backwards was quantified, which is the main contribution of this work. 

## 2. Materials and Methods

### 2.1. Powder Bed Modeling

The powder development and deposition process computation will primarily be separated into two steps: initially, a range of particles falls directly on the surface to create a powder stack; subsequently, at a defined velocity, the blade/re-coater pushes across the surface, and particles move forward into building chambers to formulate the layer.

An interaction method with the non-linear Hertz-Mindlin elastic equation is used to measure the elastic actual contact force [48], and the damping factor is theoretically applied to acknowledge the dissipation of mechanical energy [49,50,51]. 

Natural contact force and damping force in elastic materials, at which overlap between such interacting particles takes place, is always in the perpendicular plane. The relative stiffness throughout the plane is perpendicular, and Young’s modulus and mass are equal, respectively. No micro-slip approach is introduced in the tangential route to accommodate for the elastic contact force [48]. The PSD for SS316L (ERMAK-A11-S316L) provided by ERMAKSAN, Turkey, is shown in Table 1, with D10, D50, D90, and nominal range. The powder has been simulated using the given PSD. 

Throughout this research, the discrete element modeling (DEM) module from Flow Science, USA, has been used to model the layer-by-layer deposition for SS316L stainless metal powder. Rather than considering the powder layer as an equal size plate, a layer of powder was deposited using discrete microparticles. 

**Figure 1:**
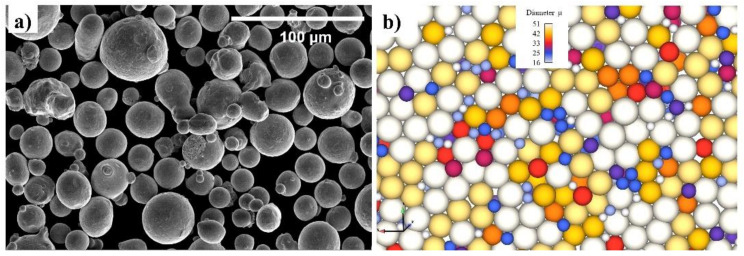
(**a**) Powder particle SEM and (**b**) discrete element modeling.

Figure 1 provides a clear picture of the powder particles. In line with the SEM picture in Figure 1a, the actual powder particle in decent approximation may be viewed as circular. The particle sizes were known to match the experiment calculation, and the particle sizes were observed to match with D10, D50, and D90, accordingly. Figure 1b displays the particle achieved through the model.

### 2.2. Modeling of Powder Bed Deposition Process

**Figure 2:**
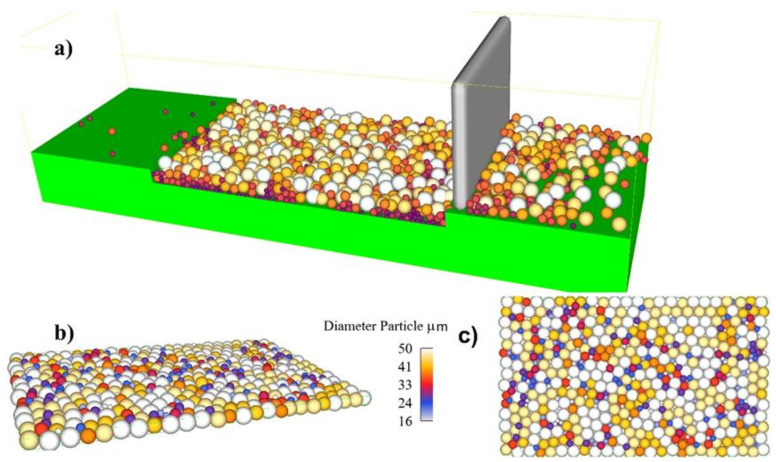
(**a**) Powder bed deposition, and (**b**) 30-micron deposited layer isometric and top view, and (**c**) side view.

Primarily based on the theoretical model mentioned above, the SS316L metal powder generation process was modeled. The powder particles’ depth of the layer is 40 μm. Figure 2 provides a clear picture of the deposition method. Figure 2a shows the DEM simulation during powder bed deposition. Figure 2b shows 30-micron deposited layer isometric and top view. A 40 μm layer was deposited in the DEM model as depicted in Figure 2a. The layer of the powder had a packing density of 65% after keeping the voids inside the block as the ones on the outer/free surface, and 90% after and eliminating the ones on the outer/free surface.

### 2.3. Modeling of Thermophysical Properties

**Figure 3:**
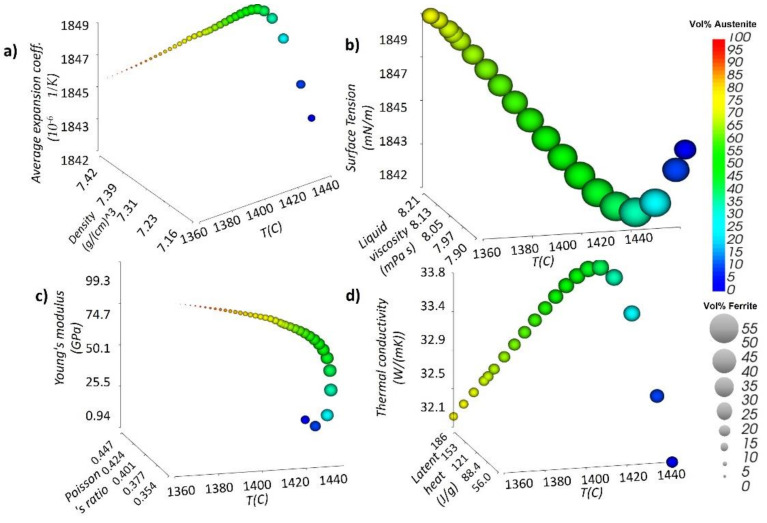
Temperature-dependent properties (**a**) density and average expansion coefficient with temperature, (**b**) Liquid viscosity and surface tension with temperature, (**c**) Poisson’s ratio and Young’s modulus with temperature (**d**) Latent heat and thermal conductivity with temperature.

For the CFD model, temperature-dependent physical properties in SS316L (ERMAK-A11-S316L) with phase changes were simulated using Sente Software, UK, based on the chemical composition (Table 2) of SS316L (ERMAK-A11-S316L) from ERMAKSAN, Turkey, as shown in Figure 3.

Based on the chemical composition provided in Table 2 of the SS316L (ERMAK-A11-S316L), the thermo-physical model has been simulated, as seen in Figure 3. Austenitic and ferritic transformation can affect the average expansion coefficient and density, as depicted in plots in Figure 3, where the sphere’s color shows the vol. % of Austenite, and the size of the sphere shows the vol. % of ferrite. Martensitic phase can also be calculated based on these two phases.

SS316L has unique behavior when compared to the other metallic alloys due to the phase complexities. The phase changes complicate the change in temperature-dependent properties. Figure 3a shows the change in liquid viscosity and surface tension concerning temperature. It can be seen that while the density is consistently decreased with temperature, the change in the average expansion coefficient (due to the phases) is not consistent, which is one of main influencing factors during the laser irradiation. Figure 3b shows the surface tension and the liquid viscosity concerning temperature [45]. It can be seen that while the temperature is consistently increasing, the change in surface tension (due to the phases) is not consistent, leading to a non-uniform Marangoni flow behavior. The Marangoni flow occurs due to the melt pool’s surface tension difference between the hot and cold end [46]. Due to the unique behavior of SS316L, the surface tension can increase at the highest temperature, which can decrease the pull in the backward direction created due to higher surface tension towards the cold end. Figure 3c shows the Poisson’s ratio and Young’s modulus, concerning temperature [45]. It can be visualized that while the temperature is consistently increasing, the change in Poisson’s ratio and Young’s moduli is not consistent. Figure 3d shows thermal conductivity and latent heat values concerning temperature, and transformed phases have also been calculated. It can be seen that while the latent heat is consistently increasing with temperature, the change in thermal conductivity (due to the phases) is not consistent, which is one of the main influencing factors for heat dissipation within the build platform [47].

### 2.4. Numerical Model

A CFD framework was designed and incorporated utilizing specific subprocesses in the FLOW-3D 11.2v CFD package and weld module from Flow Science, USA. Some of the relevant governing equations are elaborated below. The study estimates multiple factors and generalizations, for clarity: (1) the melting throughout the melt stream is considered incompressible Newtonian, and (2) the change in mass only due to metal evaporation is often not considered. 

The following equations are resolved for mass continuity, momentum, and energy conservation respectively, in Equations (1)–(3):

(1) $$\nabla \cdot \vec{v}=0$$

(2) $$\frac{\partial \vec{v}}{\partial t}+\left(\vec{\nu }\cdot \nabla \right)\vec{v}=-\frac{1}{\rho }\nabla \vec{P}+\mu \nabla^{2}\vec{v}+\vec{g}\left[1-\alpha \left(T-T_{m}\right)\right]g\left[1-\alpha \left(T-T_{m}\right)\right]$$

(3) $$\frac{\partial h}{\partial t}+\left(\vec{v}\cdot \nabla \right)h=\frac{1}{\rho }\left(\nabla \cdot k\nabla T\right)$$

where v specifies the velocity profile, $\vec{P}$ specifies pressure, μ specifies viscosity, $\vec{g}$ specifies gravity function, α specifies the coefficient of thermal expansion, ρ specifies density, h indicates specific enthalpy, while k is heat conductivity. The free surface is used to acquire the volume of fluid (VOF) in the model [52]. We can describe the VOF method as Equation (4):

(4) $$\frac{\partial V_{F}}{\partial t}+\nabla \left(\vec{\nu }\cdot V_{F}\right)=0$$

where $V_{F}$ specifies the metal volume fraction inside the cell. If $V_{F}\;$= 1, it shows that the cell is fully fluid, while $V_{F}\;$ = 0 indicates that the cell is free of the fluid. The existence of a free surface throughout this cell is shown by quantities in the center.

Factors such as thermo-physical qualities, vapor suppression, and penetration may lead to variation in melt pools. Since the Rosenthal technique is re-extracted from the heat equation and excludes evaporation, convection, and even the Marangoni impact [53,54], the corresponding term in Equation (5) for melt pool diameter is extracted from the Rosenthal formula [55] to explain the role played by thermo-physical characteristics in melting pool heterogeneity in heat transfer [53]:

(5) $$\omega =\sqrt{\frac{8}{\pi e}\cdot \frac{P\eta_{}}{\rho C_{p}V\left(T_{m}-T_{0}\right)}}$$

where ω is the melt pool width, *P* specifies beam power, η is absorptivity, ρ is density, $C_{p}$ specifies heat capacity, *V* specifies scanning velocity, $T_{m}$ specifies melting temperature, whereas $T_{0}$ specifies preheating level. The Rosenthal solution is obtained under the presumption of thermal independent physical properties as well as the thermophysical conductivity used to measure melt pool size.

The effects of recoil pressure, as well as vapor suppression on the melt pool scale, are also incorporated [56]. Each recoil pressure could be calculated by Equation (6):

(6) $$P_{S}=A\cdot exp\left\{B\left(1-\frac{T_{V}}{T}\right)\right\}$$

in which the coefficient A = β$P_{0}$, β ∈ [0.54, 0.56] and $P_{0}$ is the atmospheric pressure. The secondary coefficient *B* may be calculated as follows: *B* =$\;\Delta H_{v}$/$RT_{v}$, where $\Delta H_{v}$ was its accumulated heat of vaporization [56], *R* here is gas constant, and $T_{v}$ is the saturation temperature. *T* is now the temperature of the flow acquired through resolving the above equations [56].

The energy density of the beam is known to have a Gaussian distribution. During scanning, the beam travels at the constant scanning rate, and the ED of the beam can be represented [56] as Equation (7):

(7) $$q=\frac{2Ap}{\pi R_{b}^{2}}exp\left[-2\frac{{\left(x-\nu t-x_{0}\right)}^{2}+{\left(y-y_{0}\right)}^{2}}{R_{b}^{2}}\right]$$

where *A* specifies the powder bed’s beam absorption, p represents laser power, $R_{b}$ indicates the radius of its laser, ν is the rate of scanning, and ($x_{0}$,$\;y_{0}$) represents the laser beam center’s initial location [56]. The beam radius, $R_{b}$, is 27.5 μm. Convection, as well as radiation, was resolved upon this free surface, but evaporation cannot be ignored with the molten pool’s surface. As a result, the energy equation mostly on the surface of its molten pool may be expressed [56] as Equation (8):

(8) $$\frac{\partial T}{\partial \vec{n}}=q-h_{C}\left(T^{1}-T_{0}^{1}\right)-\sigma_{0}\varepsilon \left(T^{4}-T_{0}^{4}\right)-q_{evap}$$

where $h_{c}$ is the coefficient of convective heat transfer, $T_{0}$ is the room temperature, the Stefan–Boltzmann constant is defined by $\sigma_{0}$, ε is a measure of emissivity, and $q_{evap}$ is the heat transfer due to evaporation and could be represented [56] using Equation (9):

(9) $$q_{evap}=\omega_{0}L_{v}=exp\left(2.52+6.121-18836T-0.5logT\right)L_{v}$$

where $\omega_{0}$ is the evaporation rate. A new equation was calculated specifically for mass flow rate in this work, as calculated by Equation (10) and defined in the Results and Discussion Section.

(10) $$\dot{m}=\int \rho \cdot \vec{v}d\vec{A}$$

### 2.5. Setup for Modeling, Material Parameters, and Testing Variables

Primarily based on the theoretical framework mentioned above, the SS316L powder generation and deposition process was modeled. The bed layer size is kept at 40 μm. The implicit approach for solving convection, heat transfer, and surface voltage was used in this analysis.

To measure the temperature profile, a fixed monitoring point A inside the melt pool showed melt pool temperature [23,57] differences with respect to laser parameters. The SS316L composition is shown in Table 2, and thermal properties are dependent on the chemical composition [58].

Process variables have been selected at two different speeds (S1 = 700, S2 = 1000), with varying powers with notation S1X or S2X (where X = P1 to P6), and similarly, at two different powers (P1 = 250, P2 = 270) with varying speeds with notation P1X or P2X (where X = S1 to S6). These are used to evaluate the impact of these variables mostly on the formation of single tracks, as presented in Table 3.

### 2.6. Experimental Procedure

Single-track LPBF experiments were also performed to verify the numerical model. The single melting track was created by an ERMAKSAN ENAVISION 120, which uses the fiber laser by ERMAKSAN EON Photonics, Turkey. The machine specifications (ERMAKSAN ENAVISION 120) are listed in the Table 4. In the tests, atomized SS316L (ERMAK-A11-S316L) powder of an essentially spherical structure was used. The diameter of the beam has been kept constant at 55 μm. The air flow can be kept constant at 1 m/s to see the Marangoni flow effect clearly.

**Figure 4:**
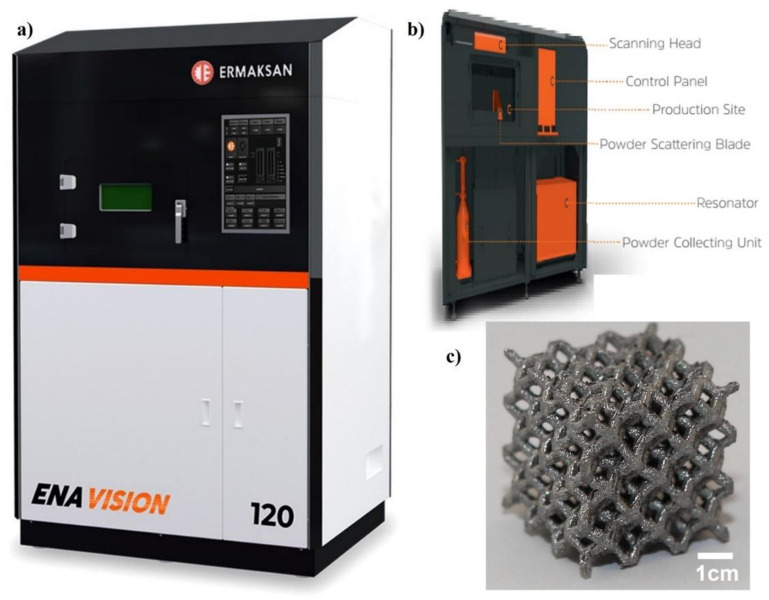
(**a**) ENAVISION 120 used for validation, (**b**) machine breakdown, and (**c**) manufactured specimen.

Figure 4a shows the ENAVISION 120 LPBF system utilized for validation, Figure 4b provides the machine breakdown, and Figure 4c shows the SS316L (ERMAK-A11-S316L) printed specimen.

FEI Nova NanoSEM 430 was used for the scanning electron microscope (SEM) micrographs. The accelerating voltage of 20 kV was used. High-voltage potential with secondary electrons is used to obtain the image. The magnification was set at 800×, the working distance was set at 8.1 mm, the horizontal field width was kept at 373 µm, and the spot size was set at 5 nm.

High-temperature Infrared Camera FLIR A655sc was used for the temperature recordings. The camera’s X direction was installed on the top of the production table and the table’s Y direction (off-axis). The camera resolution is 640 × 480 pixels and the frequency is 50 Hz. No viewing window has been used for any emission filtering. Each highest temperature value was approximated and dereived from 24 measurements, following an adjustment to reflect a consistent comparison. These values were then modified and adjusted to reflect a uniform change with power and speed of laser. The plates were cut using electrical discharge machining (EDM) for cross-sectional measurements. “Excetek” was the machine used for the tests, which has a numerical control class wire electric discharge system. It is important to keep the electrode as well as workpiece thickness consistent throughout the machining process, and the dielectric fluid utilized was water based. A digital microscope “Zeiss Smart Zoom microscope” from Carl Zeiss Microscopy Deutschland GmbH, Germany was used for optical micrographs (OM). The digital microscope uses its own software “smart zoom“ and “extending depth of field”. module used to take the 3d image.

## 3. Results and Discussion

**Figure 5:**
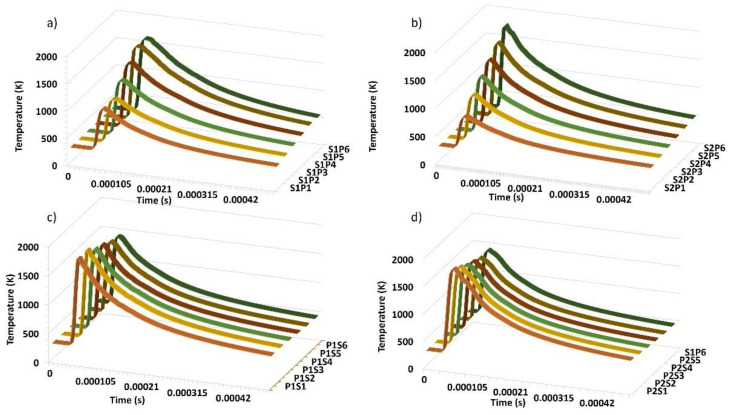
Temperature profiles: (**a**) S1P1 to S1P6, (**b**) S2P1 to S2P6, (**c**) P1S1 to P1S6, and (**d**) P2S1 to P2S6.

Through the outcome of the simulation, a probe within each single track of the layer shows the temperature profile within the melt pool (Figure 5). Figure 5a shows the temperatures reached at the 700 mm/s scanning speed with varying power from 70 to 270 W. Similarly, Figure 5b shows the temperatures reached at the 1000 mm/s scanning speed with varying power as shown from 70 to 270 W. Figure 5c shows the temperature profile at the 250 W power with varying speed from 800 to 1300 mm/s, and Figure 5d shows the temperature profile at the 270 W power with varying speed from 800 to 1300 mm/s. The difference in the melt pool temperatures shows the melting conditions.

It is clear that maximum heat input depends on the ED. By reference, the same ED means that the same amount of energy is used for a specified scanning period. ED increases with power and decreases with scanning velocity. Energy or heat dissipation also increases with the ED. The time it takes for the laser and substrate to interact determines the time it takes for the heat to be dissipated. The heat removal time is reduced at higher laser scanning rates, and the differences between different laser power and scan velocity configurations are described in Figure 5.

**Figure 6:**
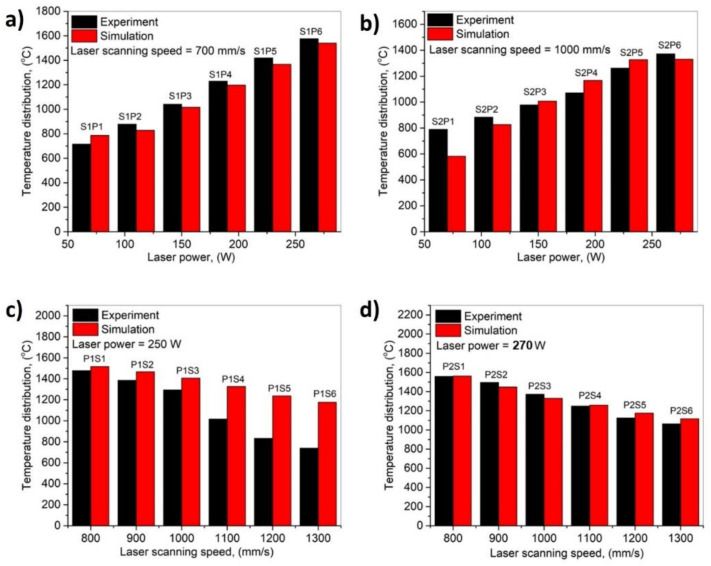
Highest temperature recording comparison with experimentation and simulation: (**a**) S1P1 to S1P6, (**b**) S2P1 to S2P6, (**c**) P1S1 to P1S6, and (**d**) P2S1 to P2S6.

The highest temperature in the simulation can also be compared with the experimental recordings in Figure 6. The simulation temperature shows a good agreement with the experimental temperature recordings. Figure 6a shows the comparison of the temperature of experimentation and simulation, at the 700 mm/s scanning speed with varying power from 70 to 270 W, Figure 6b at the 1000 mm/s scanning speed with varying power as shown from 70 to 270 W, Figure 6c at the 250 W power with varying speed from 800 to 1300 mm/s, and Figure 6d at the 270 W power with varying speed from 800 to 1300 mm/s.

When the temperature is increased from the edge (via laser), and the heat deposited surpasses the amount of heat lost, until the heat generated reaches the melting temperature followed by the formation of a pool of molten metal (this is also known as a melt-in condition in welding [59]), it is also known as the conduction mode melting in LPBF. A representative measurement was shown from the mentioned parameters.

**Figure 7:**
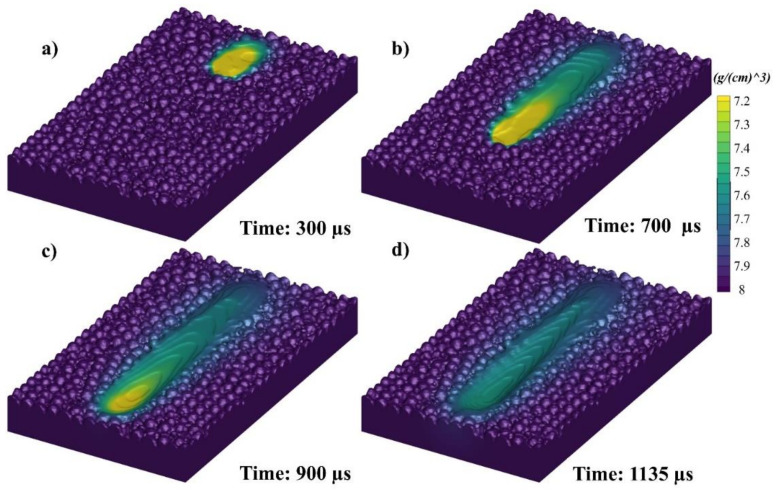
Single track melt pool profile at (**a**) 300, (**b**) 700, (**c**) 900, and (**d**) 1135 µs.

The melt pool irradiation has been elucidated with a density color gradient in Figure 7. Figure 7a–d show the melt pool at 300, 700, 900, and 1135 µs, respectively. The laser irradiates the powder bed with its velocity, and while it moves away, a temperature difference is created from the starting point to the laser irradiating end. A surface tension difference is created based on this temperature difference, which then leads to a strong Marangoni force from low surface tension towards the higher surface tension. However, another flow driving force is buoyancy, which is created due to the density within the upper and lower region of the melt pool. Both will be discussed in detail in the following section with 3D elaborations as well as in each 2D plane.

As for the unique thermal characteristics of SS316L, the surface tension can also change within the two temperature ends due to the phase changes, unlike other materials which present consistent melt pool flow. Flow anomalies discovered will also be discussed.

**Figure 8:**
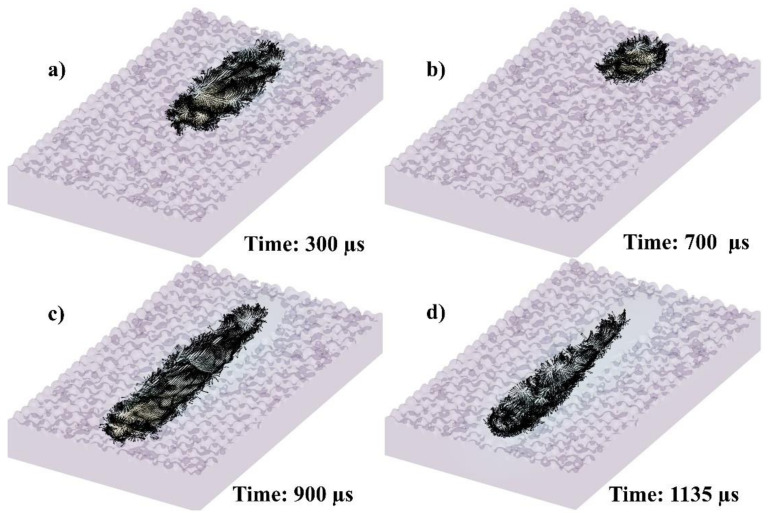
Velocity vectors of single-track melt pool in 3D at (**a**) 300, (**b**) 700, (**c**) 900, and (**d**) 1135 µs.

**Figure 9:**
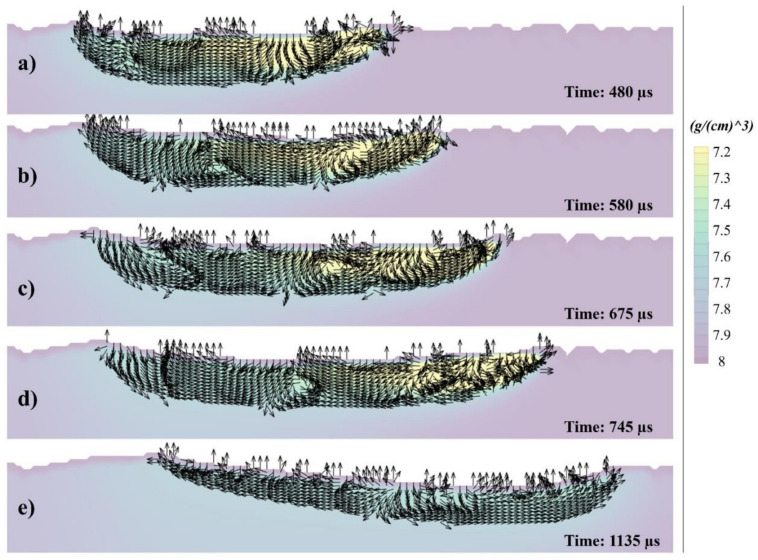
Velocity vectors of the cross-section at (**a**) 480, (**b**) 580, (**c**) 675, (**d**) 745, and (**e**) 1135 µs.

**Figure 10:**
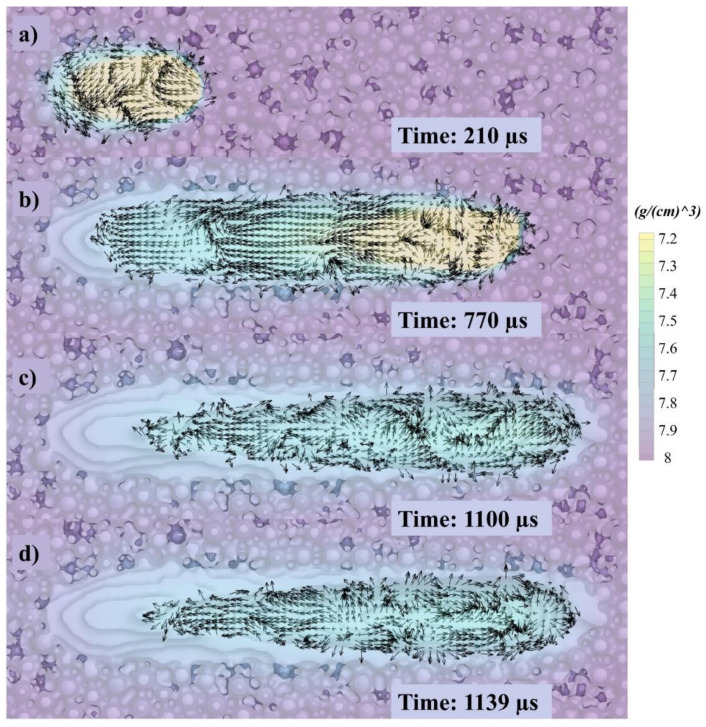
Top-view velocity vectors of single track at (**a**) 210, (**b**) 770, (**c**) 1100, and (**d**) 1139 µs.

**Figure 11:**
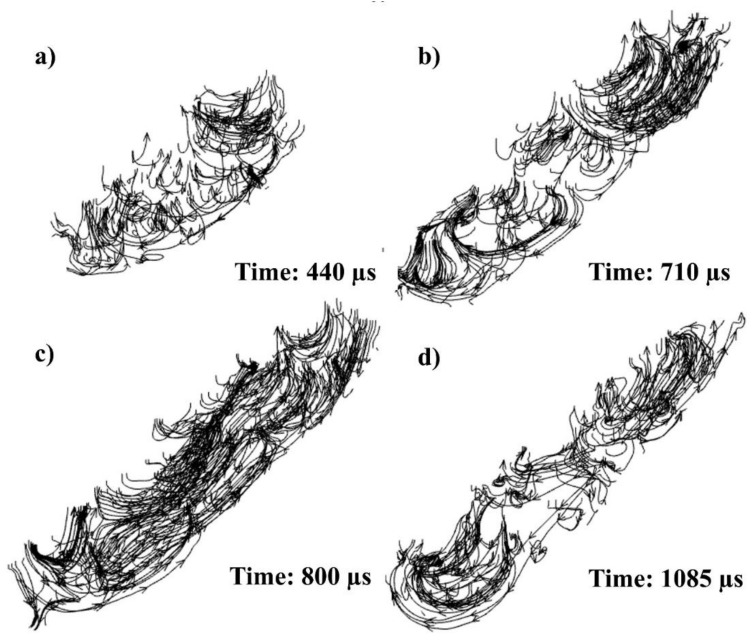
Stream traces of single track in 3D at (**a**) 440, (**b**) 710, (**c**) 800, and (**d**) 1085 µs.

**Figure 12:**
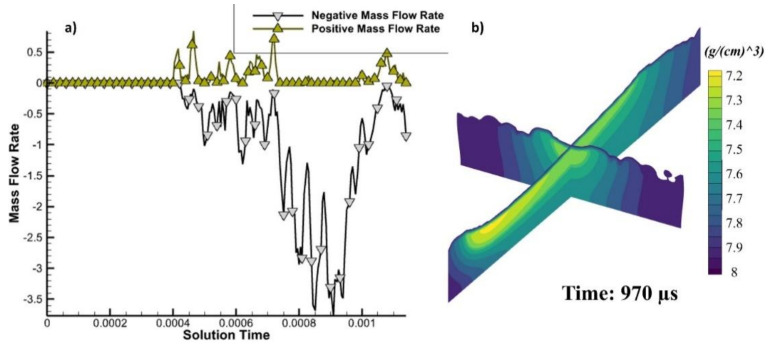
(**a**) Mass flow rate forward and backward, and (**b**) cross-section in the x-z and y-z planes at 970 µs.

**Figure 13:**
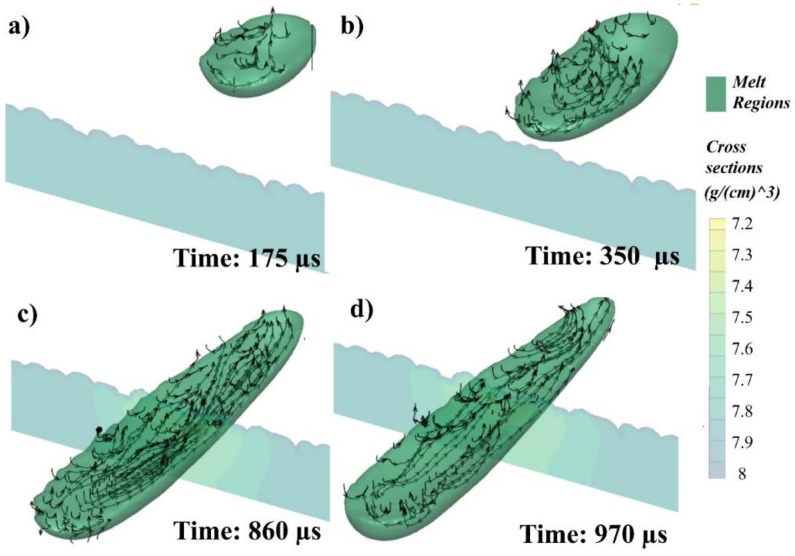
Flow passing through cross-section at (**a**) 175, (**b**) 350, (**c**) 860, and (**d**) 970 µs.

**Figure 14:**
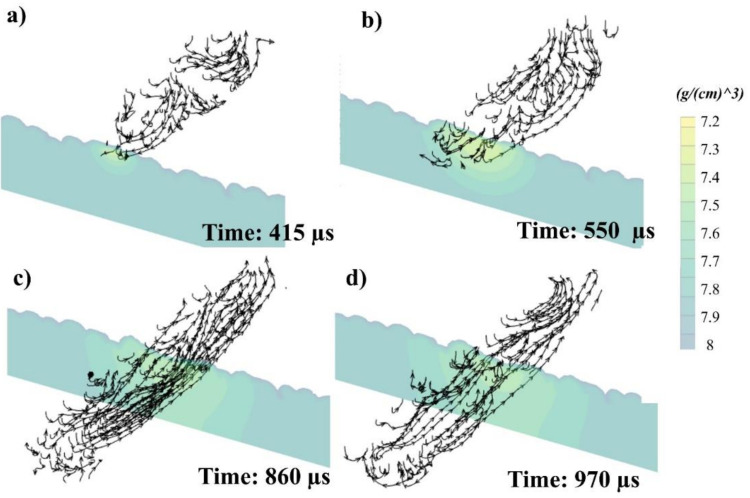
Stream traces with directions passing through the cross-section at (**a**) 415, (**b**) 550, (**c**) 860, and (**d**) 970 µs.

The melt pool velocity vectors in 3D have been calculated, as shown in Figure 8. Figure 8a–d show the velocity vectors at 300, 700, 900, and 1135 µs, respectively. The flow will further be elaborated in each 2D plane in Figure 9 and Figure 10, while the flow streams’ lines will further be elaborated in Figure 11, Figure 12, Figure 13 and Figure 14 (3D).

In SS316L, the thermal properties can cause unique flow behavior within the flow driven by Marangoni force and buoyancy. The uniqueness in SS316L is related to the austenitic and ferritic phase changes with an increase in temperature, as described in Figure 3. When the temperature in SS316L is increased over 1450 °C, the austenitic phase quickly drops, which leads to the increase in surface tension, as described in Figure 3a.

Figure 9 reveals velocity vectors of the flow within the melt pool, where the density of the melt pool is indicated by a color gradient, in the cross-sectional view, corresponding to (a) 480, (b) 580, (c) 675, (d) 745, and (e) 1135 µs. When the laser moves to irradiate the powder particles, a temperature/density gradient is created, leading to the difference in surface tension of both ends (Marangoni), as shown in Figure 9a. Due to the Marangoni force, the flow is pulled backwards [44,60,61,62]. As the laser is melting the particles, it also pushes the melt pool downwards. However, due to the density gradient from top to bottom, the newly heated metal immediately flows back to the top (buoyancy) [61,62,63,64], which can be seen in Figure 9b, where a vortex is generated.

Typically, in materials other than SS316L, the temperature gradient formed from the laser irradiating end to the rear (cool) end generates a consistent surface tension gradient [45]. However, due to the unique thermal characteristics of SS316L, the surface tension can also change within the two temperature ends triggered by ferritic and austenitic transformation, which could be the reason multiple flow directions and vortices can be generated within the melt pool, as shown in Figure 9c–e.

The flow patterns for the melt pool from the top are shown in Figure 10. In the top view, as displayed in Figure 10, arrows moving in the middle of the melt pool typically trace their movements backward; however, they are affected by the particles (small particles need minor ED and large particle need more significant ED to be heated). Therefore, a pair of cyclones is created with circulation flowing in opposing directions, as seen in Figure 10. The illuminating pendant arrows in Figure 10a show that pointers were moved from the central plane and the sidewalls in the analysis. To help visualize this concept, see Figure 10c,d, where a revolving vortex appears in the middle and towards the side walls.

Within the 3D melt pool (Figure 7 and Figure 8), the stream traces have been measured in Figure 11, showing their projectional flow. The black arrows on the upper surface of the melt exhibit the flow direction, while the stream traces within the melt indicate the flow where it initially started. In lengthwise view, flow moves from the laser irradiating region of the melt to the point where the flow initially started (rear end), which is in line with that in the transversal notion, as shown by the black arrows in Figure 10.

As the laser starts to move, the powder particles are melted with the melt pool formation and are pulled backwards due to the Marangoni force, as shown in Figure 11b, flow A. However, as the laser passes on, the melt pool can form a reverted flow due to the decrease in surface tension within the two temperature ends triggered by ferritic and austenitic transformation [65], and an opposite vortex can be formed, as shown in Figure 11b, flow B. However, it can again flow towards the back, as seen in the rear end in Figure 11b, flow C.

The flow can be divided into two parts, backwards flow (flowing towards the rear end) and forward flow (flowing towards the laser), due to the rapidness of the phenomenon and surface tension differences inherent with the unique SS316L thermal properties (Figure 11c,d). The first half (primary high-temperature Marangoni flow) shows the flow in the melt pool. In contrast, the other half (secondary lower-temperature Marangoni) depicts the flow in just the back section [61]. The 3D melt flow throughout the whole melt pool shows a wide range of flow interpretations when seen in its entirety.

A 2D cross-section was taken in the middle of the track to understand the mass flow rate, as calculated in Figure 12a. It shows the behavior of the melt pool flow during and after the laser irradiation. The positive mass flow rate shows the flow going forward, and the negative shows the flow in the backward direction. When the laser is irradiated, a bit of the melt pool flows forward, but as the laser passes that region, the melt pool cools down, and this creates a surface tension difference. The lower surface tension from the laser irradiation (hot temperature) end is pulled towards the lower temperature end (cooled region), as the fluids have very high surface tension in a cooler state. This creates a very strong backward pull, predominantly due to Marangoni flow [61]. Figure 12b shows the cross-sections after the irradiation.

Melt flow’s projectional velocity (indicated in Figure 9, Figure 10 and Figure 11) was utilized to determine the mass flow, as stated in Equation (10) and illustrated in Figure 12a. Specific directions were provided on how to demonstrate the streamlines and position, as shown in Figure 8, Figure 9, Figure 10 and Figure 11.

Note that only inside a central cross-sectional area was the flow measured (Figure 12 describes that flow speed accelerates rapidly towards the rear of that same melt pool but passes from the center). Since the velocities of the probes at various places of a flowing stream (i.e., just at the middle or towards the periphery) may vary, the overall flow streams’ speed must also vary [62]. Furthermore, the peak runoff rates are graphically shown, considering that probes in three-dimensional movement may include components that are parallel to the viewing plane. The flow speeds down the border of the melt pool and along the face are also shown in Figure 9 and Figure 10.

The aforementioned data show the flow rates in certain areas. As a consequence of the Marangoni force and buoyancy, a quantification was performed to evaluate the overall flow rate throughout the melt pool. Figure 12 clearly shows the measurement of the fluids for each region. The mass flow rate presented in Figure 12a passing through the cross-section will be clearly depicted and explained in Figure 13 and Figure 14.

The melt region of the single track passing through the cross-section with stream directions has been shown in Figure 13, where the melt pool profile can be seen at (a) 175, (b) 350, (c) 860, and (d) 970 µs. The green color is showing the melted region, while the gradient only in the cross-section shows the density. The melted region stream directions have been shown accordingly. It can be seen in Figure 13a that when the laser starts to irradiate, the particles start melting and the flow begins with a backward motion due to the laser ED inertia [61,62,63]. The melt pool starts to flow back due to Marangoni force shown in Figure 13b. There can be multiple instances where the flow can change direction (as explained in Figure 11), but the overall flow keeps moving towards the rear end, as shown in Figure 13c,d.

The streamlines’ directions of the melt pool passing through the cross-section have been separately presented more clearly in Figure 14 at (a) 415, (b) 550, (c) 860, and (d) 970 µs. It can be seen that the Marangoni flow can cause multiple cyclones caused by the surface tension differences with the melt pool triggered by the PSD of the powder, as well as the rapid irradiation of the SS316L powder (because of its phases and surface tension changes within the melt pool [45]). However, the overall flow keeps moving towards the rear end (flow rate in Figure 12).

**Figure 15:**
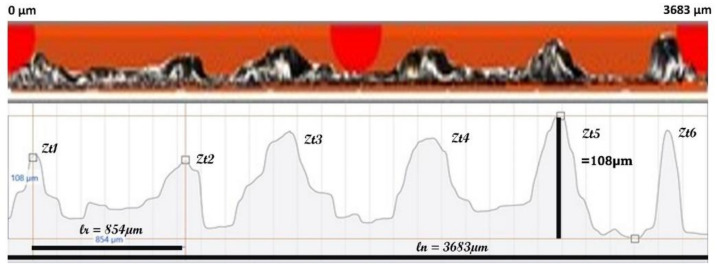
Peripheral morphology view profile of S1P1.

The single-track lateral view has been shown in Figure 15 for S1P1, where the profile can be seen. The total length of the examined region is 3683 µm. The whole region has humps caused by the melt pool mass flow migration, and the length of each hump to hump can vary from 500 to 1000 µm. It is quite consistent with the simulated results.

**Figure 16:**
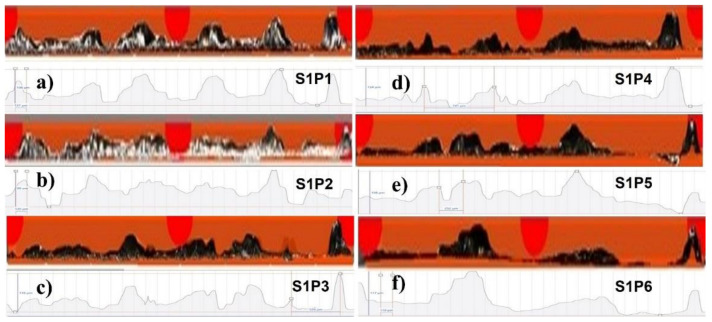
Peripheral morphology of (**a**) S1P1, (**b**) S1P2, (**c**) S1P3, (**d**) S1P4, (**e**) S1P5, and (**f**) S1P6.

Figure 16 shows the experimental micrographs in S1P1 to S1P6.

**Figure 17:**
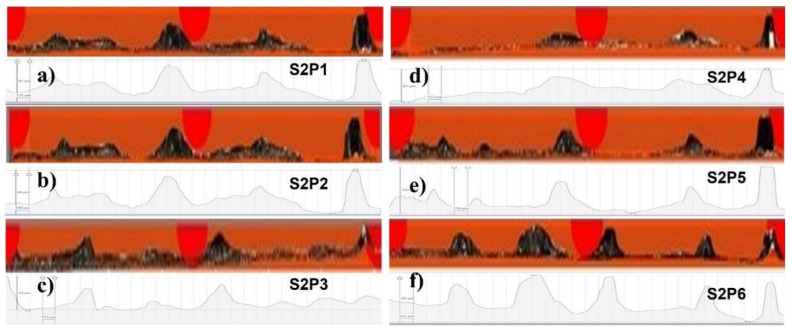
Peripheral morphology of (**a**) S2P1, (**b**) S2P2, (**c**) S2P3, (**d**) S2P4, (**e**) S2P5, and (**f**) S2P6.

**Figure 18:**
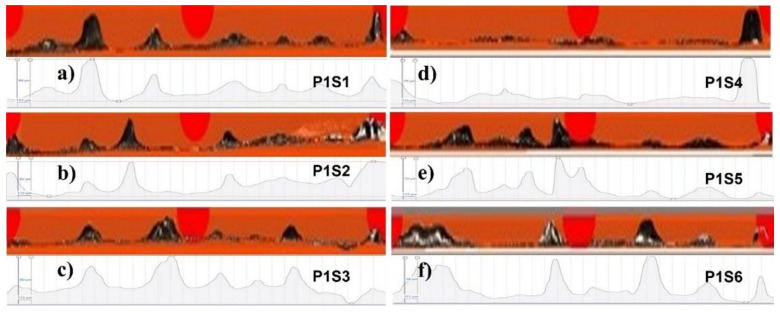
Peripheral morphology of (**a**) P1S1, (**b**) P1S2, (**c**) P1S3, (**d**) P1S4, (**e**) P1S5, and (**f**) P1S6.

**Figure 19:**
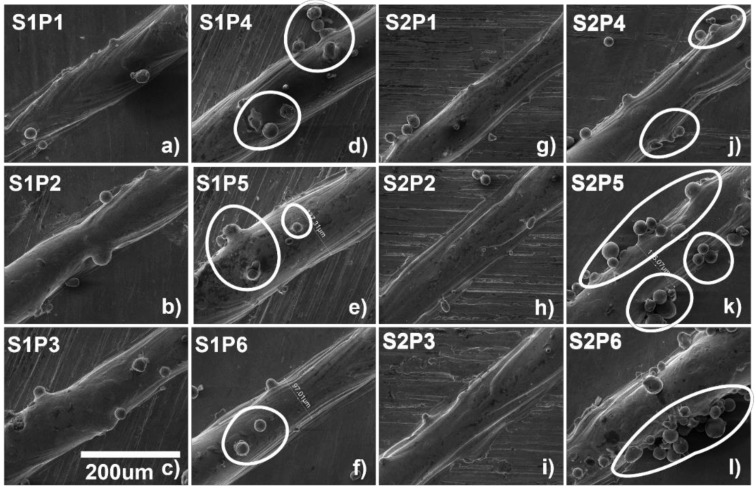
Surface morphology of single tracks for (**a**) P1S1, (**b**) P1S2, (**c**) P1S3, (**d**) P1S4, (**e**) P1S5, (**f**) P1S6, (**g**) S2P1, (**h**) S2P2, (**i**) S2P3, (**j**) S2P4, (**k**) S2P5, and (**l**) S2P6.

**Figure 20:**
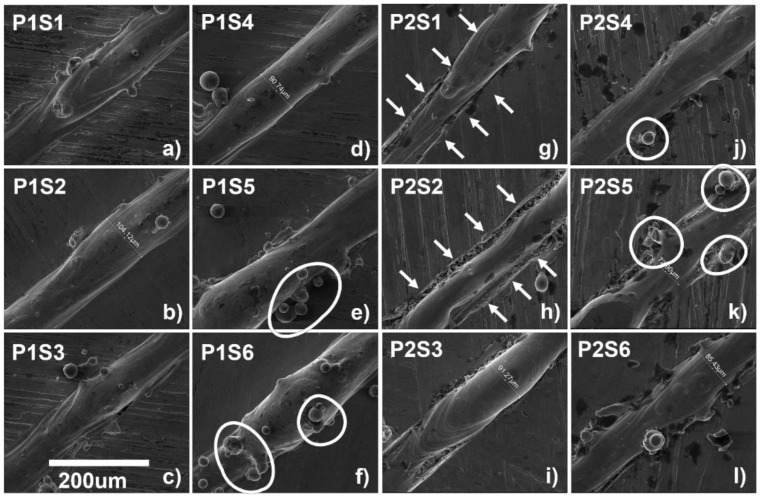
Surface morphology of single tracks for (**a**) P1S1, (**b**) P1S2, (**c**) P1S3, (**d**) P1S4, (**e**) P1S5, (**f**) P1S6, (**g**) P2S1, (**h**) P2S2, (**i**) P2S3, (**j**) P2S4, (**k**) P2S5, and (**l**) P2S6.

Similarly, Figure 17 shows S2P1 to S1P6, and Figure 18 shows P1S1 to P1S6. The profiles of the specimens at 270 W have not been presented because of the penetration in the baseplate, which will be presented later in Figure 19 and Figure 20.

As the dynamics that govern the varied melt flow trends in various locations are studied, it is possible to grasp the theoretical underpinnings that explain the observable patterns. A Marangoni force pushes flow from its elevated temperature zone to the cold temperature zone when a material has unfavorable temperature coefficients of interfacial tension.

The surface morphology of the single tracks has been presented in Figure 19, at the 700 mm/s scanning speed with increasing power from 70 to 270 W, as shown in Figure 19a–f. It can be seen that there is a consistent increase in the size of the melt pool with the increase in the laser power, which is also consistent with the simulation, similarly, as shown in Figure 19g–l at the 1000 mm/s scanning speed with varying power from 70 to 270 W. The same consistent increase in the size of the melt pool can be seen here as well.

As the ED increases, the temperature increases (as explained in Figure 5), which leads to the fusion of adjacent particles, as shown in the circular marked regions in Figure 19d–f. The same phenomena can be seen in the 1000 mm/s scanning speed shown in the circular marked regions in Figure 19j–l.

The surface morphology of the single tracks at the 250 W power with varying speeds from 800 to 1300 mm/s is presented in Figure 20a–f, and at the 270 W power with varying speed 800 to 1300 mm/s in Figure 20g–l.

It can be seen that due to the increase in speed, the inconsistency of the track increases. This is due to the temperature gradient increase with the speed (increase in Marangoni force). When the laser speed is increased, the melt pool is generated and dissipated too rapidly, leading to an increase in the temperature gradient from the rear and the front end of the melt pool [61]. While in Figure 20g,h, the base plate has also been melted due to the very high ED, this effect disappeared in the samples with higher speed and lower ED.

**Figure 21:**
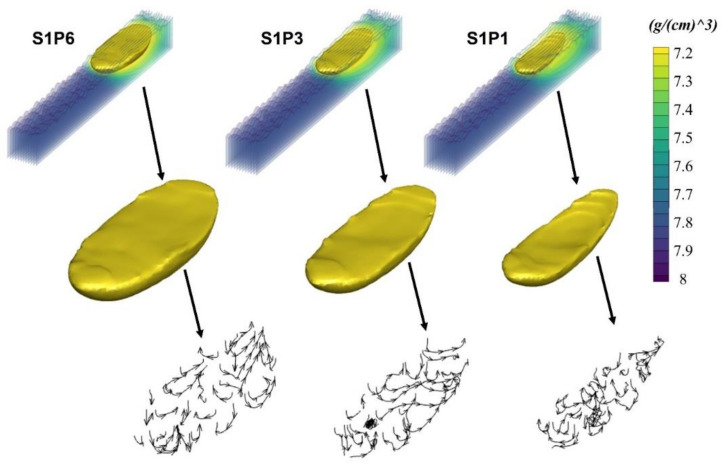
Melt pool profiles in S1P1, S1P3, and S1P6.

It can also be seen from the simulation results (good agreement with the results in Figure 21) that there is a consistent increase in the size of the melt pool with the increase in the laser power/ED, which is also consistent with the experimental findings discussed in Figure 19 and Figure 20.

**Figure 22:**
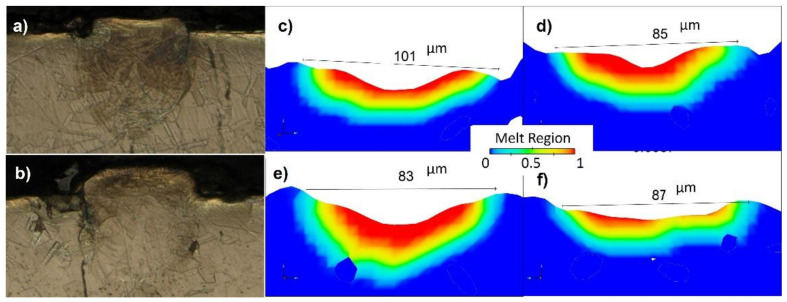
(**a**,**b**) OM at two different points of the P1S2 track. (**c**–**f**) The cross-sectional views of the same track at different points.

The formation of the melting track, which was acquired under numerous process variables, was distinct. Simulation findings revealed that different parameters, even with the same energy density (ED), can vary due to the Marangoni flow behavior. A striking phenomenon was found that at any of the specimens, the width of the melt pool was not constant, which was also validated with the optical micrograph. It could vary throughout the track for approximately 15–20 micron, e.g., the melting track at different regions of S1P1 can be seen in the OM in Figure 22a,b and in the different cross-section points of the same track simulated model in Figure 22c–f.

## 4. Conclusions

This paper has developed a DEM-based framework for simulating the process of generating powder beds and developed a 3D numerical model to evaluate the shape quality of the melt pool. By tracing evenly distributed micro-tracers via simulation, we devised a full-field melting analysis methodology to show the precise melt flow behavior within laser-additive manufacturing configurations. The computational model has been tested via experimental studies. It is necessary to draw the concluding remarks, as follows:

Due to the phase complexities, SS316L demonstrates distinct behavior as compared to other metallic alloys. The transition in temperature-based properties is complicated by phase shifts.

Once the laser is irradiated, a portion of the melt pool flows forward, but when it goes across the area, the melt pool is dragged backward driven by Marangoni flow attributed to the variations in surface tension.

The temperature profile showed a consistent increase in the highest temperature and cooling rate with the increase in power. The opposite was true for the increase in speed.

In simulations, the same melting track showed varying melt pool width due to the Marangoni force, which was also validated in the experimental results.

For the first time, we disclosed and quantified the melt flow dynamics in every spot of the whole melt pool during the melting process of LPBF. One of the important findings was that the melt flow patterns were apparent in the whole melt pool, whether the impact of the laser was apparent or not. The Marangoni effect is primarily responsible for the melt pool’s behavior. The flow pattern and flow speed are location-dependent. To obtain accurate flow speeds across the pool, the detailed flow speeds in various areas of the melt pool were measured. The mass flow rate that decreased in the irradiated zone and increased in the rear cooler region was thoroughly studied.

## Figures and Tables

**Table 1:** SS316L particle size (µm).

Nominal Range	D10	D50	D90
−45 + 15	19	30	46

**Table 2:** Chemical composition of SS316L.

Fe	Cr	Mn	Mo	Ni	Si	C	N	P	S
67.184	18	0.5	2.0	12.0	0.25	0.015	0.025	0.011	0.015

**Table 3:** Different combined process parameters.

Specimen	Scanning Speed (mm/s)	Laser Power (W)	Specimen	Laser Power (W)	Scanning Speed (mm/s)
S1P1	700	70	P1S1	250	800
S1P2		110	P1S2		900
S1P3		150	P1S3		1000
S1P4		190	P1S4		1100
S1P5		230	P1S5		1200
S1P6		270	P1S6		1300
S2P1	1000	70	P2S1	270	800
S2P2		110	P2S2		900
S2P3		150	P2S3		1000
S2P4		190	P2S4		1100
S2P5		230	P2S5		1200
S2P6		270	P2S6		1300

**Table 4:** Technical specification ERMAKSAN ENAVISION 120.

Production Volume (mm^3^)	Ø130 mm × 130 mm
Adjustable Layer Height	20–100 μm
Laser Type	Fiber Laser (Continuous Wave)
Laser Power	300 W
Scanning Speed	Up to 11 m/s (433.07 inch/s)
Scanning System	High-Speed Scan Head F-Theta Lens
Dimension (L × W × H)	1200 mm × 900 mm × 1980 mm (47.25 inch × 148.15 inch × 79.9 inch)
Electrical Connection (Voltage)	230 V, 1 PH, 50/60 Hz
Air Flow	1 to 4 m/s

## Data Availability

Not applicable.
